# Effects of chronic low-dose radiation on cataract prevalence and characterization in wild boar (*Sus scrofa*) from Fukushima, Japan

**DOI:** 10.1038/s41598-020-59734-5

**Published:** 2020-03-04

**Authors:** Samantha L. Pederson, Margaret C. Li Puma, Joshua M. Hayes, Kei Okuda, Christopher M. Reilly, James C. Beasley, Lance C. Li Puma, Thomas G. Hinton, Thomas E. Johnson, Kate S. Freeman

**Affiliations:** 1 Clinical Sciences, College of Veterinary Medicine and Biomedical Sciences, Colorado State University, Fort Collins, Colorado United States; 2 Environmental Radiological and Health Sciences, College of Veterinary Medicine and Biomedical Sciences, Colorado State University, Fort Collins, Colorado United States; 3 Institute of Environmental Radioactivity, Fukushima University, Fukushima, Japan; 4 Insight Veterinary Specialty Pathology, Davis, California United States; 5 Savannah River Ecology Laboratory and Warnell School of Forestry and Natural Resources, University of Georgia, Aiken, South Carolina United States; 6 Biomedical Sciences, College of Veterinary Medicine and Biomedical Sciences, Colorado State University, Fort Collins, Colorado United States

**Keywords:** Physiology, Environmental impact, Anatomy, Biological physics

## Abstract

This study evaluated cataracts in wild boar exposed to chronic low-dose radiation. We examined wild boar from within and outside the Fukushima Exclusion Zone for nuclear, cortical, and posterior subcapsular (PSC) cataracts *in vivo* and photographically. Plausible upper-bound, lifetime radiation dose for each boar was estimated from radioactivity levels in each animal’s home range combined with tissue concentrations of ^134+137^Cesium. Fifteen exposed and twenty control boar were evaluated. There were no significant differences in overall prevalence or score for cortical or PSC cataracts between exposed and control animals. Nuclear (centrally located) cataracts were significantly more prevalent in exposed boar (*p* < 0.05) and had statistically higher median scores. Plausible upper-bound, lifetime radiation dose ranged from 1 to 1,600 mGy in exposed animals, with no correlation between dose and cortical or PSC score. While radiation dose and nuclear score were positively associated, the impact of age could not be completely separated from the relationship. Additionally, the clinical significance of even the highest scoring nuclear cataract was negligible. Based on the population sampled, wild boar in the Fukushima Exclusion Zone do not have a significantly higher prevalence or risk of cortical or PSC cataracts compared to control animals.

## Introduction

The lens of the eye (intraocular lens) is one of the most sensitive tissues to ionizing radiation^1,2^, making cataracts an excellent indicator of long-term tissue effects from low-dose radiation exposure. Radiation-associated cataracts have been documented in non-human animals, airline pilots, astronauts, medical radiation technologists, radiation-treated human and canine cancer patients, Chernobyl clean-up workers, and atomic-bomb survivors^3–7^. The visual impact of cataracts ranges from inconsequential to blinding, with the latter requiring surgical intervention to restore sight.

Radiation-induced cataractogenesis is currently considered a deterministic effect, where biological changes occur once a certain dose threshold is reached^8^. The historical threshold for cataract development was two Gray (Gy)^9,10^, but has recently been lowered to 0.5 Gy^11^. Now, some researchers question whether a threshold even exists for cataracts, or their development is a stochastic effect rather than deterministic, creating debate amongst scientists and radiological protection organizations^1,12–15^.

Cataracts, opacifications within the intraocular lens, can form in different locations, including the nucleus (center of the lens), cortex (outer layer), and the posterior subcapsular space (back of the lens, just inside the membranous capsule). Among these, only cortical and posterior subcapsular (PSC) cataracts have consistently been related to ionizing radiation; nuclear cataracts (referring to the anatomic location within lens rather than the etiology) have only infrequently been associated with radiation^4–6,16–22^. Additionally, there are no known characteristics or biomarkers to determine the specific etiology of a cataract once it has formed, adding difficulty to retrospective cataract evaluations.

Appropriate assessment of cataract development requires detailed information regarding cataract type, location, and score or severity. Therefore, it is critical for ophthalmology-trained scientists to be involved in cataract evaluations. It is also imperative to understand differences in terminology and agree upon a mutual use of language. The LOCS (lens opacity classification system) compares opacifications within the lens to a set of standard images, providing a score for lesions within different parts of the lens. This system was developed in an attempt to standardize cataract evaluations and is now commonplace in clinical and research settings. Although there is no defined threshold at which an ‘opacity’ becomes a ‘cataract’, some authors set a cut-off to help establish clinical relevance^23^. And while other advanced imaging modalities now provide more objective means of cataract evaluation, LOCS is universally available, usable in a field setting, and requires no special equipment^24,25^. Modalities such as histology are also useful for identifying cataractous changes within the lens, but require sampling of the exact cross-section of the lesion, making this an impractical survey method.

Ophthalmic expertise has been previously lacking in studies assessing radiation-associated cataracts in free-ranging wildlife^26,27^. For example, a study in Chernobyl small mammals detected cataracts in 71% of bank voles (*Myodes glareolus*) sampled^27^; however, there was no control population and no cataract localization or characterization^26,27^. In addition, post-mortem and freezing artifacts may have influenced the appearance of cataracts^28–32^. A similar evaluation in avian species^33^ also lacked ophthalmologist involvement and incorrectly identified cataracts as opacities that may be obscuring the visibility of the iris, an ocular structure anatomically located in front of the lens.

Despite the intraocular lens being an effective indicator of chronic radiation exposure, there are only a handful of studies evaluating the ocular effects of low-dose exposures in humans, and a single study in mammalian wildlife^17,19,27^. This is likely due to the inherent difficulty in replicating or identifying environments with relevant exposures, as well as numerous potential confounding factors associated with *in situ* experiments that are difficult to control. Chen *et al*. studied cataracts in 114 Taiwanese people following ~15 years of chronic exposure to radiation from cobalt-60 incorporated into building materials^19^. When evaluating individuals exposed to >5 millisieverts (mSv) of cobalt-60 per year, a significant dose-dependent increase in the number of cataracts was observed in individuals <20 years of age compared to those 20–40 years and ≥40 years. Similarly, Day *et al*. studied over 1,700 Ukrainian children, ages 5–17 years, following the 1986 Chernobyl nuclear accident^17^. Although their vision was clinically unaffected, exposed children had significantly more PSC cataracts than non-exposed children^17^.

Extensive environmental release of radionuclides occurred from the Fukushima Dai-ichi nuclear power plant after the Great East Japan Earthquake and Tsunami of March 2011. Following the accident, ~165,000 people were evacuated from a 20 (and then 30)-kilometer radius surrounding the reactors to minimize human exposure to radiation^34^. Much of the area has now been remediated and humans are slowly returning. However, the most contaminated portion, the ‘difficult to return zone’ or Fukushima Exclusion Zone (FEZ), remains void of human residents due to persistently high radiation levels. Since the accident, multiple studies have evaluated radio-cesium levels in human residents from cities neighboring the evacuated area^35–37^. Although initial studies detected internal radioactive cesium in up to 35% of individuals, nearly all doses were <1 mSv^35^. Dose rates in peripheral regions of the FEZ have dropped precipitously since 2011, and portions of the FEZ have been deemed ‘returnable’^38^. While some residents have elected to return to their homes, many remain concerned about their exposure to low levels of radiation and the resultant risks. It is therefore imperative to use biological models, such as wild boar, living in contaminated areas to assess the potential safety risks for returning humans.

Following human evacuation, many animals, including wild boar (*Sus scrofa*), opportunistically inhabited the developed, and now contaminated, human environments^39^. Wild boar are an ideal sentinel species for monitoring low-dose rate radiation effects on the intraocular lens due to multiple factors. First, boar have similar ocular anatomy, physiology, and lens protein composition to humans^40,41^. Secondly, both humans and boar are omnivores, and an adult boar has a body mass similar to humans (~50–100 kg^42^), although their quadrupedal posture creates an increased surface area exposed to the contaminated ground. Further, global positioning system dosimetry collars^43^ attached to free-ranging wild boar within the FEZ revealed that boar can be exposed to dose rates exceeding 50 μGy/hr. And although some differences in weight and size exist between populations on differing continents, wild boar from the United States, Europe, and Japan are all of the same species, *Sus scrofa*, with no known physiologic differences in cataract susceptibility^44,45^.

The purpose of this study was to evaluate the prevalence and characterization of cataracts in wild boar chronically exposed to radiation within the FEZ relative to a control population. We hypothesized to find significant differences in cortical and PSC cataracts between groups, with exposed boar having a higher cataract prevalence, as well as overall higher cataract scores according to the Lens Opacity Classification System (LOCS) III^46^. We also hypothesized cataract scores would correlate positively with plausible upper-bound lifetime radiation dose in exposed animals.

## Results

**Figure 1:**
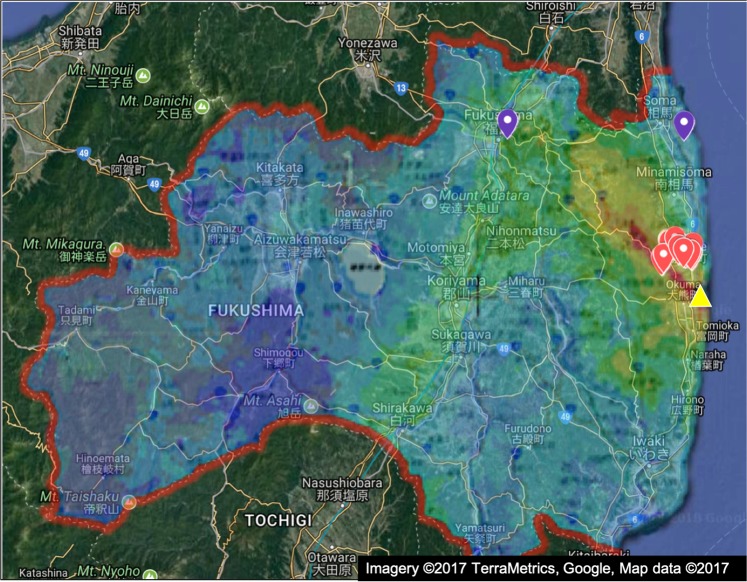
Map of the Fukushima Prefecture, with added colored overlay of the air radiation dispersion (northwest from the Fukushima Daiichi Nuclear Power Plant [yellow triangle], located on the coast, due east of Okuma-town) documented in November 2011. Highest levels of contamination are noted in red (>19 μSv/h) followed by orange (9.5–19 μSv/h), yellow (3.8–9.5 μSv/h), and green (1.0–3.8 μSv/h). Dose rates were measured 1 m above the ground surface. Red markers indicate trapping sites of exposed boar. Purple markers indicate trapping sites of Japanese control boar. Traps were specifically set in higher-contaminated regions and home ranges are generally around 10 km^2^, thus boar trapped in the yellow/orange zones are likely to travel within the red zone as well. (Google 2017; Extension Site of Distribution Map of Radiation Dose, etc./GSI Maps).

Thirty eyes from 15 wild boar within the FEZ and four eyes from two boar outside the FEZ were evaluated from Japan. (Fig. 1) Due to a lack of sufficient control animals collected from Japan, 36 eyes of 18 wild pigs from uncontaminated areas of the Savannah River Site (SRS) in Aiken, South Carolina were evaluated. This resulted in a total of 15 exposed and 20 control animals. Following plausible upper-bound lifetime radiation dose estimation, both ‘control’ boar from Japan were determined to have significant total body radiation doses. Therefore, these animals were excluded from ‘control’ vs. ‘exposed’ comparisons but retained in analyses investigating cataract score relative to total lifetime radiation dose.

The median age of boar for both exposed and control groups was 62 weeks (range: 26 to >220 weeks) based on tooth eruption patterns^47^ (Table 1). A total of 23 males and 12 females were evaluated with nine males and six females in the exposed group, and 13 males and five females in the control group after excluding the one male and one female from the control group in Japan with elevated radio-cesium levels (Table 1). Exposed and control groups were comparable in terms of age (*p* = 0.53) and sex (*p* = 0.49). The median weight for all animals was 46.5 kg (22.5 to 88.6 kg) and the median weight of control boar was significantly higher than exposed boar (56.8 kg vs. 39.4 kg, respectively; *p* = 0.02). One control boar was diagnosed with a congenitally abnormal right eye, as demonstrated by peripheral anterior synechiae, lens coloboma, posterior cortical cataract, and a persistent hyaloid artery. Due to the multiple congenital abnormalities, this eye was removed from the study.

**Table 1:** Demographic attributes of wild boar (*Sus scrofa*) sampled from the Fukushima Exclusion Zone, Japan (exposed) and the Savannah River Site, USA (control) from 2017–2018 for cataract assessment.

	Exposed (n = 15 boar) median (range)		Control (n = 18 boar)^†^ median (range)		*P* value
Age (weeks)	62 (26 to >220)		62 (26 to >220)		0.53
Weight (kg)	39.4 (22.5–77.9)		56.8 (34.1–88.6)		0.02*
Sex	**Male**	**Female**	**Male**	**Female**	
	9	6	13	5	0.49

Age was determined by tooth eruption and wear patterns. Weight was acquired by a field scale once animals were immobilized. Mann-Whitney test was used to compare age and weight; Fisher’s Exact test was used to compare sex between groups.

*Significant *p* < 0.05.

^†^Excluding 1 male and 1 female ‘control’ from outside the FEZ in Japan, but with elevated total body radiation doses.

### Anterior segment diagnostics and examination findings

There were no significant differences between right (OD; oculus dextrus) and left (OS; oculus sinister) eyes of exposed or control animals for tear production as measured by Schirmer Tear Test (*p* = 0.65 OD vs. OS of exposed boar; *p* = 0.64 OD vs. OS of control boar; *p* = 0.10 exposed vs. control boar) or fluorescein stain indicating corneal ulceration (positive in 3/30 eyes of exposed boar, 1/33 eyes of control boar; Table 2). One boar did not have fluorescein stain performed in either eye and the congenitally abnormal eye was not included. There was no difference between OD and OS for intraocular pressure within exposed and control groups (*p* = 0.89 OD vs. OS of exposed boar; *p* = 0.64 OD vs. OS of control boar); however, median intraocular pressure was significantly higher in exposed boar compared to control animals (11.5 vs. 9.5 mmHg; *p* = 0.02) (Table 2).

**Table 2:** Anterior segment diagnostics for 30 exposed and 35 control eyes collected during 2017–2018 from wild boar (*Sus scrofa*) from the Fukushima Exclusion Zone, Japan and the Savannah River Site, USA, respectively.

	Exposed (n = 30 eyes) median (IQR)	Control (n = 35 eyes) median (IQR)	Range	*P* value
Schirmer Tear Test (mm/min)	7 (6–12)	6 (4.25–9)	0–16 mm/min	0.10
Intraocular Pressure (mmHg)	11.5 (8.75–13.25)	9.5 (7.25–11)	4–24 mmHg	0.02*
Fluorescein Stain (+)	3/30 eyes^†^	1/33 eyes^‡^		

One eye from a control boar was excluded due to congenital abnormalities; all other animals had both eyes included. Mann-Whitney test was used to compare Schirmer tear test and intraocular pressure between exposed and control animals. The Schirmer tear test was not expected to be normal as animals were anesthetized (domestic swine normal values are 15.6 ± 3.7 mm/minute^95^). Intraocular pressure was significantly higher in the exposed group than the control, suspected to be due to lower anesthetic depth. Fluorescein stain was applied to corneal surface for detection of corneal ulcerations.

*Significant *p* < 0.05.

^†^One ulcer suspected to be iatrogenic during enucleation.

^‡^Two eyes of one control animal not stained.

One exposed boar had prominent keratitis (corneal inflammation) with a stromal facet (healed corneal ulcer) and eyelid entropion (inward rolling of the eyelid margin) OD. Four control boar had anterior chamber abnormalities, including posterior synechiae (adhesions of iris to lens capsule), iris cysts, and free pigment on the anterior lens capsule.

### *In vivo* cataract evaluation: lens opacity classification system (LOCS) III

Nearly all animals had visible suture lines (‘Y-shaped’ connections between lens fibers at the anterior and posterior aspects of the lens). These were considered a normal anatomic finding within the lens and not a cataract. Mittendorf’s dots (embryologic remnants of the hyaloid vascular system) were also often visible as a punctate opacity on the ventral aspect of the posterior lens capsule and were not considered a cataract. For the purposes of this study, the term ‘cataract’ was used to identify an opacity within the lens as demonstrated by the LOCS III and did not distinguish a visually impairing cataract from a non-visually impairing one (*see Methods for further details*).

Spearman r values for nuclear (centrally located), cortical, and PSC cataracts between *in vivo* observers (SLP, MCL) were 0.66, 0.80, and 0.82, respectively. All comparisons were significant (*p* < 0.0001), indicating acceptable (0.66) to good (0.80, 0.82) correlation between *in vivo* observers. Limits of agreement were (−0.47 to 0.39) for nuclear scores, (−0.32 to 0.38) for cortical scores, and (−0.07 to 0.08) for PSC scores. Therefore, values from a single observer (SLP) were used for all evaluations.

**Figure 2:**
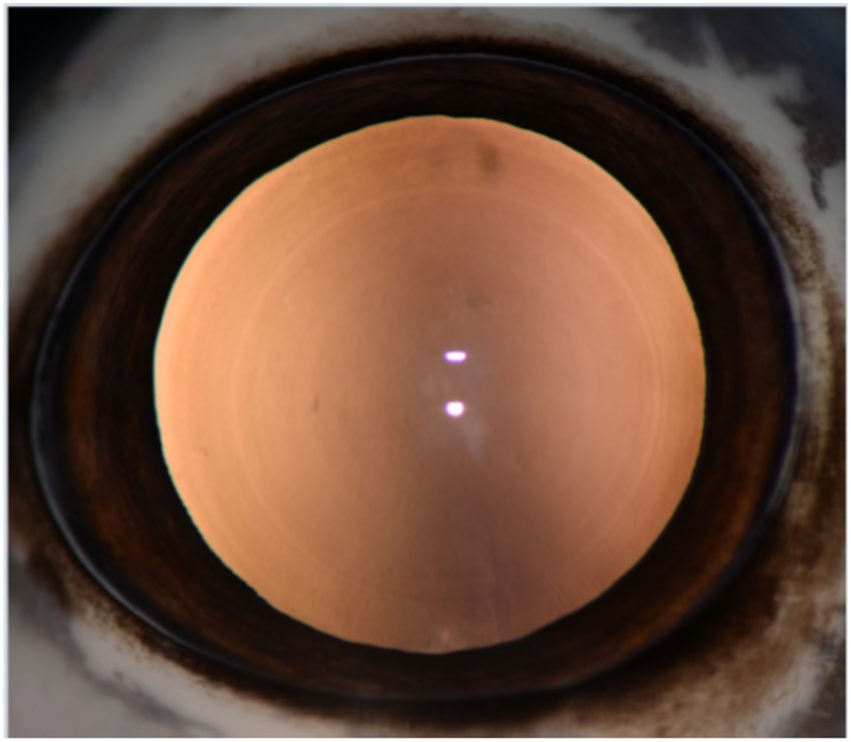
Normal lens in the right eye of a Japanese wild boar (*Sus scrofa*) from the Fukushima Exclusion Zone seen on retroillumination. Note two vertically-aligned circular reflections (flash artifact) on the central corneal surface. Vertical central shadow is artifact from the slit lamp positioning in front of the camera and flash. Subtle concentric rings are visible, representing the normal separations between layers of the lens.

**Figure 3:**
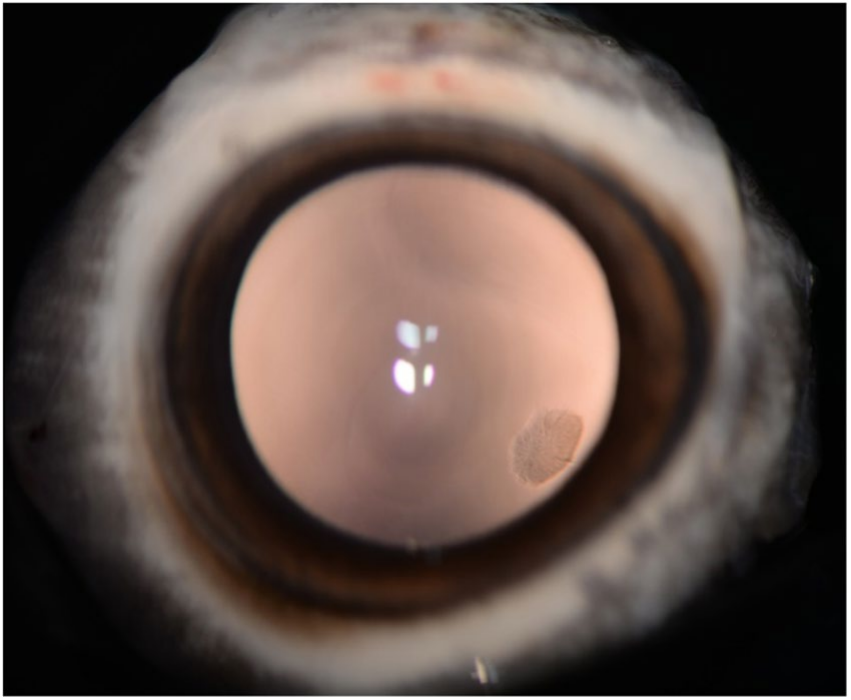
Posterior subcapsular cataract at 4 o’clock in the lens of the left eye of a Japanese wild boar (*Sus scrofa*) from the Fukushima Exclusion Zone seen on retroillumination. Note four vertically-aligned circular reflections (flash artifact) on the central corneal surface. Lens Opacity Classification System III PSC score 2.4, 2.7, 2.0 (MCL, SLP, KSF).

**Figure 4:**
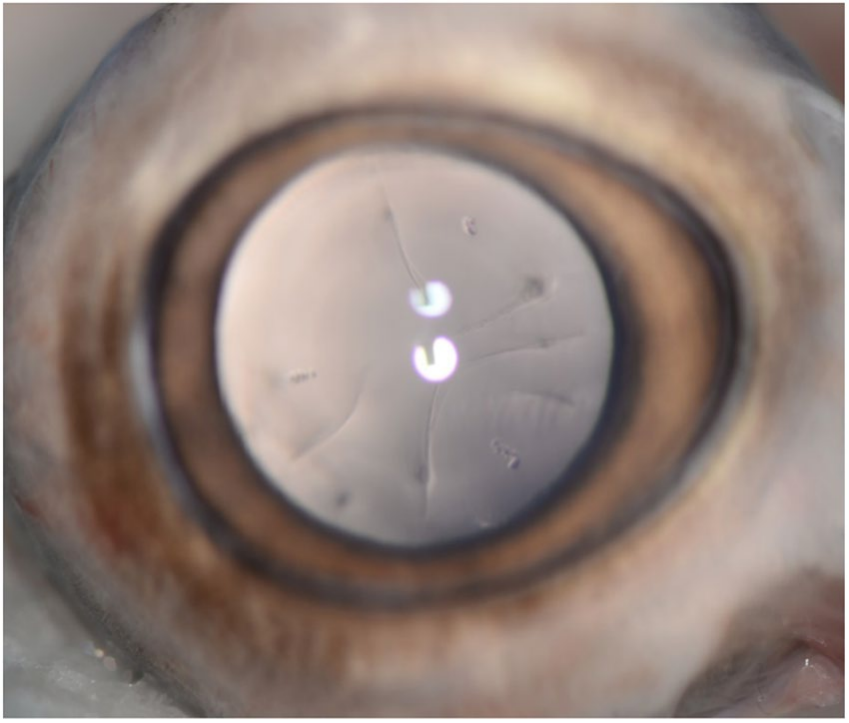
Cortical cataract (peripheral dots and radiating lines, commonly described as ‘spoke-wheel’ appearance) in the lens of the left eye of a control wild pig from the Savannah River Site seen on retroillumination. Due to the depth of the opacities, the iris becomes unfocused in order to focus on the posterior cortex of the lens (anatomically located behind the iris). Note two vertically-aligned circular reflections (flash artifact) on the central corneal surface. Lens Opacity Classification System III cortical score 1.4, 2.3, 2.4 (MCL, SLP, KSF).

There were no significant differences in overall *in vivo* cataract prevalence for cortical (*p* = 0.12) or PSC (*p* = 0.24) cataracts between exposed and control boar. Lens Opacity Classification System III scores were also not significantly different between exposed and control animals for cortical (*p* = 0.14) or PSC (*p* = 0.21) cataracts (Tables 3 and 4). The LOCS III scale begins at 0.1 (no visible cataract; Fig. 2) and increases by increments of 0.1 up to 5.9 (cortical, PSC) or 6.9 (nuclear) based on cataract type in comparison to a set of standard images. Cortical scores ranged from 0.1 (no visible cataract) to 1.2 for exposed animals (median 0.1) and 0.1 to 2.3 for control animals (median 0.1; Fig. 3). Posterior subcapsular scores ranged from 0.1 to 2.7 for exposed animals (median 0.1; Fig. 4) and 0.1 to 0.2 for control animals (median 0.1). When evaluating the influence of plausible upper-bound lifetime radiation dose (Table 5) on cataract score, no relationship was found between dose and cortical (*p* = 0.08; R^2^ = 0.02) or PSC (*p* = 0.16; R^2^ = 0.002) score (Table 5).

**Table 3:** Lens Opacity Classification System III scores of wild boar (*Sus scrofa*) collected during 2017–2018 from the Fukushima Exclusion Zone, Japan (exposed) and the Savannah River Site, USA (control).

	LOCS III Score	Exposed (n = 30 eyes)	Control (n = 35 eyes)	*P* value
**Nuclear opacity**	Grade ≤0.1	2	9	
	Grade >0.1	28	26	>0.05
**Cortical opacity**	Grade ≤0.1	27	26	
	Grade >0.1	3	9	0.12
**PSC opacity**	Grade ≤0.1	28	37	
	Grade >0.1	2	0	0.24

By LOCS III, cataracts are compared to a standard set of photographs and scored on a decimal scale by increments of 0.1. Nuclear opacities are scored from 0.1–6.9 (0.1 no cataract, 6.9 most severe cataract), while cortical and PSC opacities are scored from 0.1–5.9 (0.1 no cataract, 5.9 most severe cataract). Number of exposed and control eyes are listed for each cataract type based on scores of ≤0.1 or >0.1. Mann-Whitney test was used to compare cataract presence vs. absence in control vs. exposed animals for each cataract type. Both eyes from each animal were included, except the congenitally abnormal eye from a control boar.

LOCS III, Lens Opacity Classification System III; PSC, posterior subcapsular.

*Significant *p* < 0.05.

**Table 4:** Lens Opacity Classification System III scores of wild boar (*Sus scrofa*) collected during 2017–2018 from the Fukushima Exclusion Zone, Japan (exposed) and the Savannah River Site, USA (control).

	LOCS III Score	Exposed (n = 30 eyes)	Control (n = 35 eyes)	*P* value
**Nuclear opacity**	Grade ≤1	25	35	
	Grade >1	5	0	0.005*
**Cortical opacity**	Grade ≤1	29	34	
	Grade >1	1	1	>0.99
**PSC opacity**	Grade ≤1	29	35	
	Grade >1	1	0	0.49

Number of exposed and control eyes are listed for each cataract type based on scores ≤1 and >1 to provide a more clinically relevant breakdown of Table 3. Common LOCS III values to be considered ‘cataracts’ in humans are ≥ 2 or 4 for nuclear and ≥ 2 for cortical or PSC opacities^23,62^. Mann-Whitney test was used to compare cataract score (≤1 or >1) in control vs. exposed animals. Both eyes from each animal were included, except the congenitally abnormal eye from a control boar.

LOCS III, Lens Opacity Classification System III; PSC, posterior subcapsular.

*Significant *p* < 0.05.

**Table 5:** Plausible upper-bound lifetime radiation dose (see Supplementary information for dose determination methods) and cataract scores for wild boar (*Sus scrofa*) sampled from the Fukushima Exclusion Zone, Japan in 2017. Nuclear scores are based on a decimal scale of 0.1–6.9 (0.1 no cataract, 6.9 most severe cataract); cortical and PSC scores are based on a scale of 0.1–5.9 (0.1 no cataract, 5.9 most severe cataract).

Boar Identification	Estimated Age (weeks)	Plausible Upper-bound Lifetime Dose (mGy)^a^	Highest Nuclear score	Highest Cortical score	Highest PSC score
Ba20170605	26	333	0.3	0.1	0.1
Ba20170608	30	90	0.1	0.1	0.1
Ba20170609	>220	1596	1.4	0.1	0.1
Bb20170609	47–52	338	1.2	0.1	0.1
Ba20170616	47–52	338	0.2	0.1	0.1
Bb20170616	62	41	0.8	0.1	0.1
Ba20170620	62	433	0.8	0.2	0.1
Ba20170623	127	413	0.8	0.2	0.1
Bb20170623	88–106	861	1.3	0.1	0.1
Ba20170627	62	433	0.3	0.1	0.1
Ba20170704	56–62	419	0.4	0.1	2.7
Ba20170717	57–61	407	0.3	1.2	0.9
Bb20170717	127	863	0.2	0.1	0.1
Bc20170717	127	860	0.7	0.1	0.1
Ba20170724	62	422	0.3	0.1	0.1
Ba20170615^*^	>220	42	1.5	0.1	0.1
Ba20170617^†^	26	1	0.4	0.1	0.1

The highest scoring eye for each cataract type was used for each animal.

^a^See Supplementary information.

^*^Ba20170715 trapped within a ‘control’ site, but ambient dose was 0.46 µSv/hr (significantly higher than expected).

^†^Other ‘control’ animal from Japan; ambient air dose <0.1 µSv/hr.

When evaluating the presence of any detectable cataract (LOCS III score >0.1), no difference in nuclear cataract prevalence was present between groups. However, when evaluating cataract presence based on a LOCS III score >1.0, exposed animals had a significantly higher prevalence of nuclear cataracts than control animals. Additionally, exposed boar had significantly higher nuclear scores (median 0.35) compared to controls (median 0.2; *p* < 0.001) and a significant association was found between plausible upper-bound lifetime radiation dose and nuclear score (*p* < 0.0001; R^2^ = 0.48).

Although exposed boar had higher nuclear scores, we also observed a significant positive association between age and nuclear score (*p* < 0.005; R^2^ = 0.43); no association was observed between age and cortical or PSC score. However, age was also correlated with total lifetime radiation dose (R^2^ = 0.48), suggesting our observation of higher nuclear scores in more contaminated animals was confounded by age.

### Plausible upper-bound, lifetime dose to individual boar (Supplementary information)

Supplementary information provides details on the methodology used to estimate plausible upper-bound lifetime dose to individual boar. Original calculations were performed based on values from Harshman *et al*. using electron spin resonance (ESR) dosimetry^48^. All but one sample was below the method’s detection limit (DL), giving confidence that the upper-bound dose to which the boar were exposed was <1.8 Gy. The large number of samples below the ESR detection limit prevented a thorough statistical analysis of cataract prevalence and severity as a function of chronic radiation exposure. Thus, estimates to each boar were also based on external dose from the animal’s contaminated environment, combined with internal dose from ^134+137^Cs activity concentrations in each animal’s muscle tissue. This method resulted in plausible upper-bound lifetime doses that ranged from 1 to 1596 mGy (Table 5 and Supplementary Table S1).

### *Ex vivo* Cataract evaluation: photographic and ImageJ analysis

Spearman r values comparing cataract scores between *in vivo* observer (SLP) and *ex vivo* photographic evaluations (KSF) for nuclear, cortical, and PSC scores were 0.10, 0.43 and 0.59, respectively. These comparisons for cortical and PSC scores were significantly correlated (*p* < 0.0005 and *p* < 0.0001, respectively), but nuclear scores were not (*p* = 0.41). Limits of agreement were (−0.58 to 0.86) for nuclear scores, (−0.55 to 0.55) for cortical scores, and (−0.23 to 0.27) for PSC scores. *In vivo* observations indicated a difference in nuclear scores between exposed and control animals, although this was not supported by photographic evaluations. Software analysis of nuclear scores using ImageJ (ImageJ, NIH, Bethesda, MD, USA) revealed pixel intensities ranging from 29.4–145.4. Photographic evaluation of the nucleus was heavily impacted by photographic settings and image brightness, altering the photographic interpretation of nuclear scores as well as the pixel intensities. Therefore, only *in vivo* values were used for nuclear scores. While photographic variability was unlikely to impact the detection of cortical or PSC cataracts, it likely had a significant impact on nuclear scoring, explaining the difference in nuclear scores between *in vivo* observer and *ex vivo* photographic evaluation. Ultimately, nuclear cataracts have not been strongly associated with radiation exposure (as cortical and PSCs have), so the impact on overall outcome was deemed minimal.

Wisconsin grading was performed on retroilluminated images for any cortical or PSC cataracts visualized, and grades were compared between *in vivo* and photographic evaluators. *In vivo* exams provided Wisconsin grades for 11 eyes (three exposed, eight control). Photographic exams provided Wisconsin grades for five eyes (three exposed, two control). Three eyes (one exposed, two control) were scored by both *in vivo* and photographic evaluations, including both cortical and PSC cataracts (Table 6). Two of the shared eyes had LOCS III scores >2; the third eye was graded as 0.9 *in vivo* and 1.5 by photographic evaluation for a cortical cataract.

**Table 6:** Wisconsin Scores for the one wild boar (*Sus scrofa*) and two wild pig eyes scored in both *in vivo* examination and photographic evaluation.

	*In Vivo* Wisconsin Score	Photographic Wisconsin Score
Ba20170704 OS^*^	2B, 3A center	2B
Bc20180109 OD^†^	3B, 4A	3A, 4A
Bc20180109 OS^†^	1B, 2A, 3B,4A	1A, 1B, 2A, 2B, 3A, 3B

The *in vivo* examiner scored an additional eight eyes and photographic examiner scored an additional two eyes. Overall, there was good correlation and agreement between *in vivo* and photographic scores for cortical and PSC cataracts (Spearman r values of 0.43 and 0.59, respectively; limits of agreement (−0.55 to 0.55) and (−0.23 to 0.27), respectively). Nuclear cataracts are not graded on the Wisconsin system.

*Wild boar from Fukushima Exclusion Zone, Japan.

^†^Wild pigs from Savannah River Site, USA.

### Histopathology

**Figure 5:**
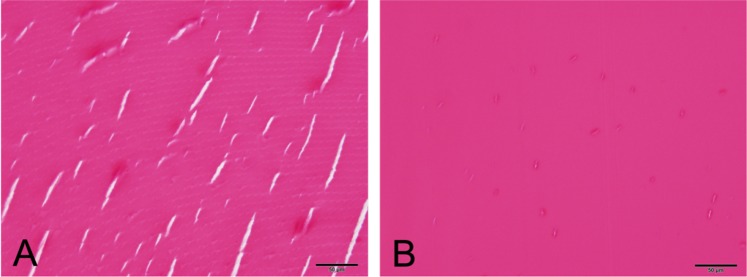
Photomicrographs of the lens nucleus of wild boar. (**A**) Normal nuclear clefts. Note vague, curved lamellations. Artifactual clear (white) clefts are largely parallel and geometric. H&E. Bar = 50 *μ*m. (**B**) Lens hyalinization. Note the lack of fiber definition and homogeneous, glassy (hyalinized) appearance. Artifactual clear (white) clefts are small and rarely scattered. H&E. Bar = 50 *μ*m.

Due to the inherent difficulties in detecting cataracts histologically and the high degree of type II error, only the degree of nuclear hyalinization (a degradative change of the lens fibers) was objectively evaluated (Fig. 5). When compared to *in vivo* nuclear score, no correlation was found (*p* = 0.78; r = 0.05).

**Figure 6:**
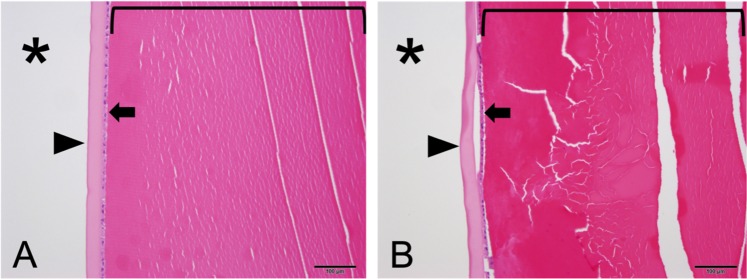
Photomicrographs of the anterior lens of wild boar. (**A**) Normal anterior lens. Note a relatively orderly, lamellar pattern to the fibers (bracket), and artifactual clear (white) clefts are geometric and largely parallel. (**B**) Anterior cortical cataract. The fibers of the cortex (bracket) lack lamellar definition, are more deeply eosinophilic (red), and clear (white) clefts are irregularly oriented and often curvilinear. * = anterior chamber; arrowhead = lens capsule; arrow = lens epithelial cells. H&E, Bar = 100 *μ*m.

The PSC cataract in Fig. 3 was detected histopathologically. No other cataracts detected *in vivo* were noted histologically. Additionally, two lenses were noted to have cortical fiber swelling and disorganization (Fig. 6), with no *in vivo* or photographic detection of cataract.

## Discussion

This is the first published study characterizing cataracts in large, free-ranging wild mammals exposed to chronic low doses of environmental radiation. Ultimately, no significant differences were seen in prevalence or severity of cortical or PSC cataracts (most commonly associated with radiation) in exposed wild boar compared to control animals. While previous studies have attempted cataract evaluation in small mammals and birds^27,33^, this study offers a comprehensive scientific evaluation of the anatomic location and quantitative degree of cataracts, along with detailed plausible maximum lifetime radiation dose (Supplementary information).

No differences were observed for cortical or PSC cataracts, but significant differences were found for nuclear (within the central lens) cataract scores between exposed and control animals. However, nuclear cataracts are also the cataract type least consistently associated with radiation^49,50^. The intraocular lens is comprised of an outer basement membrane (capsule), inner anterior layer of epithelial cells, and central lens fibers. The most central and oldest fibers comprise the ‘nucleus’ while the younger, more external fibers comprise the ‘cortex’. Lens epithelial cells continually replicate and migrate posteriorly throughout the life of an animal, elongating, losing organelles, and becoming more transparent and fiber-like^51,52^. While the particular mechanisms of radiation-induced cataracts remain to be completely elucidated, it is generally thought that damage to the peripheral, active lens epithelial cells (germinative zone) by any toxic insult results in abnormal maturation of the metamorphosing lens fiber^53^. This makes it unlikely for toxic insults, such as radiation, to affect the oldest, innermost fibers within the nucleus of the lens.

In this study population, minimal predictive power was also added by including age as a confounder to the relationship between total lifetime radiation dose and nuclear score. The data indicate that as animals age, both nuclear score and total body radiation dose increase. This is not surprising, as the lens nucleus becomes denser with age and the older an animal is, the more cumulative radiation is acquired. It is therefore difficult to completely separate the effect of age versus total body radiation dose on nuclear score, and the clinical impact of our findings for nuclear cataracts is reduced.

The narrow age range of wild boar in this study and variability between ages of exposed vs. control animals may also have influenced the results and should be taken into consideration. The lack of correlation between radiation exposure and cortical or PSC cataracts in this study may largely be due to a study population of animals that were too young to have shown the radiation-induced cataract effects. The maximum lifespan of a wild boar can reach 9–10 years, yet in the wild most live a few years or less and those that make it to maturity typically do not live past 5 years^54^. The age of boar in this study ranged from 26 to >220 weeks (0.5–4.2 years), with only 2 animals >150 weeks (2.9 years), representing only a fraction of the potential lifespan; however, these samples reflect the natural distribution of boar ages observed in the Fukushima population. Depending on the age of the lens and type of toxic insult, DNA defects within lens epithelial cells may also produce latent cataracts months to years following the exposure^55,56^. It has been shown that the young, juvenile lens is generally more sensitive than an adult lens^17,19,57^, and the latent period of cataracts may also be related to the lens age at the time of exposure^58^. Latency period is also affected by fractionation, the act of dividing a single large radiation dose into multiple smaller doses^59,60^. This should be considered when evaluating the long-term effects of low radiation doses, as the cataract latency period may be longer than a short-lived species’ life expectancy and there is likely large species variability in latency of cataract development itself.

It is also clinically important to note that none of the opacities found in this study were considered vision-impairing cataracts (VICs)^61^. The highest scored PSC cataract was 2.7 *in vivo* and 2.0 photographically (Fig. 3), while the highest scored cortical cataract was 2.3 *in vivo* and 2.4 photographically (Fig. 4). The results from this study also show good agreement for cortical and PSC cataract identification between both *in vivo* observers and *ex vivo* photographic analysis. While the authors are unaware of a universally agreed upon specific criteria for VICs, common cut-offs for ‘cataracts’ using the LOCS III classification (nuclear cataracts ≥2 or 4, cortical or PSC cataracts ≥2) indicated no boar were actually diagnosed with nuclear cataracts, and only one exposed boar and one control boar was diagnosed with a PSC and cortical cataract, respectively^23,62–67^. The highest *in vivo* and *ex vivo* photographic nuclear scores were 1.5 and 0.5, respectively. Evaluation of humans have only found significant improvements in maximum reading speeds after surgical removal of nuclear cataracts with LOCS III scores ≥5^68^, suggesting any nuclear cataracts in boar from this study had minimal impact on their vision. This also demonstrates the minimal clinical significance of the difference in median nuclear score between exposed and control animals (0.35 vs. 0.2). Furthermore, that which constitutes a ‘nuclear cataract’ in human ophthalmology would often still be considered ‘nuclear sclerosis’ or ‘lenticular sclerosis’ in veterinary ophthalmology, and not undergo surgical intervention. This difference demonstrates the varying clinical impacts and importance of transparent terminology. Future cataract studies in wildlife species should include clear differentiation of the location and grade of these lesions, due to the significant differences in their clinical impact and outcomes.

The other significant ophthalmic finding was a difference in intraocular pressures between exposed and control animals (Table 2). While intraocular pressure can be lowered secondary to chronic inflammation from significant cataracts, this is unlikely as the values for both groups were within normal range. Additionally, even though the values are statistically different, the clinical difference of 2 mmHg between groups is negligible. We suspect this difference was due to a lower depth of anesthesia in exposed animals. Several animals also had mild corneal and eyelid disease, or evidence of intraocular disease (chronic inflammatory changes). Two animals with these intraocular changes had cataracts and two did not. It is most likely that a systemic illness or ocular trauma resulted in cataract formation rather than radiation exposure. However, as there is no retrospective method to identify the etiology of cataract formation, this cannot be confirmed. One boar estimated to be >220 weeks had significant entropion (inward rolling of the eyelid margin) in one eye, most likely secondary to facial trauma.

As with any study based on biological sequelae from radiation exposure, there is inherent difficulty in interpretation of raw data and results. Cataractogenesis in particular can be impacted by several factors^19,69,70^. Some of these, such as smoking or the topical and/or systemic use of corticosteroids^71–73^, are not applicable to wild animals, while others like diet, ultraviolet ray exposure^63,74,75^, and systemic disease^71,76^, may play a role in cataract development in wildlife. Ultraviolet ray exposure is a large confounder for the development of cataracts, so our control population was specifically chosen from a geographic location with similar sun exposure and elevation to Fukushima, Japan. Historical large-scale chronic low-dose exposure studies utilizing natural or man-made radiation disasters encounter similar difficulties^19,77,78^. Studies based on environmental radiation from man-made reasons (Techa River Study^79,80^) are often limited by appropriate long-term follow up, and those based on medical radiation exposures are biased by the study population receiving therapy or working in the at-risk field (US Medical Radiological Technicians Study^81,82^; CT Diagnostic Scans Study^83^). Studies from workers in the nuclear industry (Mayak Worker Study^84,85^; 15-Country Study of Nuclear Workers^86^; US Shipyard Workers Study^87^; Study of Radiation Workers at DOE Sites^88,89^) are also limited by the age of subjects at the time of exposure and several confounding demographic and lifestyle factors. Nearly all of the studies struggle with inconsistent cataract scoring, low statistical power to detect small risks and effects, and uncertainties in dose estimates^77,78,90^.

Limitations to this study include *in vivo* ophthalmic examinations and photographs performed in a field setting, estimation of animal age, low sample size, predominantly young animals, and differences in geography between exposed and control populations. While a significant association was found between total body dose and nuclear score, the young age of subjects likely distorted/skewed the relationship. Scheimpflug imaging is a diagnostic modality used to more objectively evaluate cataracts – however, this requires a table-mounted device and an animal with a flat face and relatively small nose, which are impractical for the field setting as well as for use *in vivo* with boar. Further, although objective grading scales utilized in human ophthalmology were used for cataract scoring, the scales remain somewhat subjective. And while a board-certified veterinary ophthalmologist evaluated lens photographs, this individual was not present for *in vivo* examinations.

This study provides the most comprehensive evaluation of cataracts in free-ranging wildlife exposed to chronic radiation to date. In particular, identification of cataracts within specific locations of the lens by multiple trained observers has not previously been performed in wild animals^27,33^. And while determining radiation dose to free-ranging animals has considerable uncertainties, our method of estimating plausible lifetime radiation dose adds significant strength to the study (Supplementary information). Finally, this study demonstrates the importance of multidisciplinary collaborative efforts between radiation ecologists, veterinary ophthalmologists, wildlife ecologists, and health physicists^22^.

## Conclusions

Wild boar within the Fukushima Exclusion Zone did not have a significantly higher prevalence or score of cortical or PSC cataracts compared to control animals. While cortical and PSC cataracts were found in both control and exposed animals, none of the cataracts were considered visually-impairing, even with estimated plausible upper-bound lifetime radiation doses up to 1.6 Gy. Nuclear (centrally located) cataracts had a higher prevalence and median score in exposed vs. control boar, but age could not be completely removed as a confounder. Based on historical studies and the overall inconsistent association between nuclear cataracts and radiation, we expect the relationship found is this study is most likely due to age.

Although the sample size is low, this study provides valuable data on the negligible impact of 1–4 years of chronic low dose radiation on cataract formation in wild boar, a model species for cataract formation in humans. In line with the NCRP’s desire for ‘high-quality epidemiological and mechanistic studies’^1^, cooperative and collaborative research on sentinel wildlife species can lend significant contributions to better understanding environmental and human health impacts from low-dose radiation exposures.

## Methods

### Ethics Statements

No animals were killed specifically for this research. All animal use was secondary to, and in collaboration with, government sponsored culling programs to control wildlife pests. Animal use methods were in compliance with the Association for Research in Vision and Ophthalmology statement for the Use of Animals in Ophthalmic and Vision Research and approved by Colorado State University and University of Georgia Institutional Animal Care and Use Committees, IACUC 17–7080A and IACUC A2015 05–004-Y3-A6, respectively.

### Animals

Wild boar (*Sus scrofa leucomystax*) were obtained from within the Fukushima Prefecture, Japan during June and July 2017. Boar were opportunistically live-trapped in wire-mesh cages by government-sponsored hunters. Exposed boar were trapped at various locations within the FEZ and uncontaminated control boar were trapped outside the FEZ. Due to low trapping success of control animals in Japan, additional controls were trapped on the Savannah River Site (SRS) in Aiken, South Carolina, USA during January 2018 by experienced wildlife biologists. Wild pigs on the SRS consist of hybridized wild boar and feral pigs and were deemed more appropriate controls over local domestic swine due to similarities in genetic composition, morphology^42,44^, geographic habitat, and elevation.

The study design preferentially selected adult boar over juveniles by excluding animals initially estimated to be <20–25 kg. Age was determined by tooth eruption and wear patterns by an experienced wildlife biologist (KO)^47^.

### Anesthesia and Euthanasia

#### Japan

Trapped boar were anesthetized with intramuscular 5 mg/kg Zoletil (reconstituted to 100 mg/ml, equal concentrations of tiletamine and zolazepam; Zoletil 100, Virbac, Australia) and 0.1 mg/kg medetomidine (10 mg/ml, Domitor) via pole dart (DAN-INJECT, Denmark), based on estimated weight^43^. A dark tarp and canopy were placed around the cage to minimize visual and auditory stimulation prior to and during anesthesia. If animals failed to show signs of sedation within 3–5 minutes, a full repeat dose was administered. Anesthetic depth was determined by lack of voluntary movement upon physical stimulation. Following an appropriate anesthetic depth, the muzzle was taped, and animals were weighed using a field scale. After 10–15 minutes, every boar received an additional IV (subclavian) top-up dose for researcher safety (same dose but based on actual weight instead of estimated), as well as immediately prior to euthanasia.

#### SRS

Trapped wild pigs were anesthetized with intramuscular 4.4 mg/kg Telazol (reconstituted to 250 mg/mL, equal concentrations of tiletamine and zolazepam; Telazol; Zoetis, US) and 0.1 mg/kg medetomidine (20 mg/mL, compounded) via dart gun (Pneu-Dart, Inc., Williamsport, PA) based on estimated weight. If darts did not embed within the musculature and animals were not showing signs of sedation within 3–5 minutes, a full repeat dose was administered. If boar were anesthetized longer than 30 minutes or began exhibiting signs of consciousness (increases in respiration, voluntary muscle movement), a top-up dose was administered (1/2 the initial dose). Darting doses for SRS pigs were lower due to improved drug delivery with the dart gun versus pole dart. Top-up dose was also lower due to overall improved anesthetic plane with better drug delivery from the first injection.

### Ophthalmic examination

Tear production was evaluated via Schirmer Tear Test I (STT) while eyelashes were trimmed to ease *in vivo* ophthalmic examination and enucleation. Anticholinergic mydriatics (1% atropine ophthalmic solution and 1% tropicamide ophthalmic solution) were instilled onto each globe for pupillary dilation, followed by rebound tonometry (TonoVet, iCare, ‘d’ setting; Paragon Medical, Coral Springs, FL) to evaluate intraocular pressure. Slit lamp biomicroscopy of adnexal and anterior segment structures (SL-17; Kowa, Germany) was performed under a dark cloth to decrease background light. Lens evaluation occurred following subjectively appropriate pupillary dilation.

Two examiners (SLP, MCL) recorded independent observations according to two different cataract quantification systems to allow for complete lens opacity characterization: the Lens Opacity Classification System III (LOCS III) and the Wisconsin System^46,91,92^. The LOCS III system identifies cataracts within the nucleus, cortex, and posterior subcapsular (PSC) space of the lens and scores them based on a standard set of images, but does not account for the x-y location. Nuclear cataracts are graded from 0.1–5.9; cortical and PSC cataracts are graded from 0.1–6.9. Specifically, a nuclear cataract was an increase in nucleus density compared to the surrounding cortex; a cortical or PSC cataract obscured light, creating a white lesion on transillumination and a black shadow on retroillumination.

The Wisconsin System utilizes a circular grid to map opacities on an x-y axis but does not account for the anterior-posterior axis. Additionally, this system does not quantify nuclear (centrally located) cataracts. Indirect fundoscopy was performed with a binocular headset and 28 diopter double aspheric lens (Volk Optical, Mentor OH, USA). Abnormalities were documented using a ClearView fundic camera (Optibrand, Fort Collins CO, USA).

### Blood collection, euthanasia, and necropsy

Following complete ophthalmic examination, blood was collected from the subclavian artery and animals were euthanized via exsanguination, so as not to interfere with cataract evaluation or contaminate the environment. Death was confirmed by lack of heartbeat and absent corneal reflex^93^.

Following euthanasia, a general physical exam, including standard measurements (weight, body length, longitudinal distance, hock length, ear length), note of any external injuries or pathologies, and on-site necropsy were performed. Globes were promptly enucleated via subconjunctival surgical method. If no cataract was identified, one eye was randomly selected (coin toss) for dissection and lens weight, to be utilized in a parallel study. If cataract or significant anterior segment abnormalities were identified, that globe was preferentially selected for complete histopathology and the contralateral globe was dissected for lens weight. Globes were evaluated with fluorescein stain (FUL-GLO® fluorescein sodium ophthalmic strips USP, Akorn, Lake Forest, IL, USA) for corneal ulcerations prior to placement in formalin.

Globes were placed in 10% neutral buffered formalin for fixation^94^. The intact globe was moved to 70% ethanol after seven days for storage and transport. A board-certified ocular pathologist (CMR), masked to all exam findings and radiation exposures, evaluated globes for ocular histopathology.

Mandibles were removed and photographed for age determination based on tooth eruption and wear patterns^47^. Muscle tissue (*Biceps femoris*) samples were homogenized and measured for radio-cesium concentrations using a high-purity germanium detector (HPGe; GC3018 Canbera Japan, Tokyo) for estimating internal radiation dose (Supplementary information).

### Photographic lens evaluation

Immediately following enucleation, globes were photographed in a darkened space. Slit beam images were obtained with an iPhone6 and Camera + application (LateNiteSoft 2016, Madrid, Spain), while holding the 0.1 mm SL-17 slit beam at a 45-degree angle on the globe. The cornea was continuously irrigated with 0.9% saline to maintain a smooth and clear optical surface. Slit beam videos were taken for the majority of the eyes to provide additional evaluation of any abnormalities.

Retroilluminated images were obtained with the diffuse beam of the SL-17 directed to create a fundic reflection, and a Nikon 660 DSLR with 105 mm macrolens with a focus of 0.314–0.37, ISO settings of 200–800, F-stop 4.8–5, and shutter speed of 60–160. Lens photographs were quantitatively evaluated for cataracts using ImageJ 1.50i computer software, the LOCS III, and the Wisconsin System by a board-certified veterinary ophthalmologist (KSF), masked to *in vivo* findings and radiation exposure^91,95^.

### Radioactivity measurements and estimation of dose (Supplementary information)

Plausible upper-bound lifetime dose (Table 5; Supplementary Table S1) was determined for each boar using radioactivity levels specific to each animal’s home range combined with its internal body radioactivity concentrations. Plausible upper-bound lifetime doses were correlated with cataract prevalence and score for each boar within the FEZ.

### Statistical analyses

Calculations from previous radiation-induced cataract studies in controlled rat models were used to predict sample size. Data indicated 15 subjects per cohort were sufficient to detect a 20% difference in cataract prevalence between exposed and control eyes, accounting for at least 80% power (β = 0.2) and α = 0.05^67^.

Statistical software RStudio (version 3.2.4, http://www.rstudio.com/) and GraphPad Prism (version 8.1.0 for macOS, GraphPad Software, La Jolla, California USA, www.graphpad.com) were used to perform analyses. Spearman rank test and Bland-Altman analysis were performed to examine correlation and agreement between *in vivo* examiners and each *in vivo* examiner with the photographic evaluations (SLP vs. MCL; SLP vs. KSF; MCL vs. KSF). Relationships between cataracts and dose were evaluated in two ways: (1) Comparison of cataract prevalence (present vs. absent) and cataract score in exposed versus control animals, and (2) Comparison of highest cataract score for each subtype (nuclear, cortical, PSC) to plausible upper-bound lifetime radiation dose. Each boar was categorized according to the eye with the highest cataract score for each location (nuclear, cortical, PSC).

All data was evaluated for normality using a Shapiro-Wilk Test and determined to be non-normally distributed. Therefore, results are presented using the median and interquartile range for continuous data. Categorical data are presented as absolute values. A Fisher’s Exact Test was performed for categorical values (sex and cataract presence/absence) while the Mann-Whitney Test was used for non-parametric comparisons of continuous data (age, weight, Schirmer tear test, intraocular pressure, and cataract score). Correlation and linear regression were performed to evaluate the relationship between plausible upper-bound lifetime dose and cataract score, as well as for nuclear score and degree of nuclear hyalinization, as determined by histopathologic evaluation. Multiple linear regression was used to evaluate the interactions between age, radiation dose and cataract score.

## Supplementary information

Supplementary information.

## Data Availability

The datasets generated during and/or analyzed during the current study are available from the corresponding author upon reasonable request.
