# Supplementary material for: Effects of chronic low-dose radiation on cataract prevalence and characterization in wild boar (Sus scrofa) from Fukushima, Japan
Source: Sci Rep. 2020 Mar 4;10:4055. doi: 10.1038/s41598-020-59734-5 (PMC7055243; doi:10.1038/s41598-020-59734-5)
Supplement: Supplementary file 1 — Supplementary information. [file 41598_2020_59734_MOESM1_ESM.docx]

**SUPPLEMENTARY INFORMATION: Methods to Estimate Lifetime Radiation Dose**

**Effects of chronic low-dose radiation on cataract prevalence and characterization in wild boar (*Sus scrofa*) from Fukushima, Japan**

Samantha L Pederson, Margaret C Li Puma, Joshua M Hayes, Kei Okuda, Chris M Reilly, James C Beasley, Lance C Li Puma, Thomas G Hinton, Thomas E Johnson, Kate S Freeman

Cataract formation is most likely a function of an animal’s lifetime dose, rather than the detectable dose at time of capture. However, it is challenging to determine the dose to a free-ranging animal due to of the heterogeneity of contamination in the animal’s environment. Extrapolation to lifetime dose is even more difficult with larger uncertainties. Therefore, this supplement describes the methods of determining plausible upper-bound lifetime doses to the wild boar used in the cataract analyses.

**METHODS**

Original dose calculations were performed using Electron Spin Resonance (ESR) dosimetry values determined by Harshman *et al.*^1^. Electron Spin Resonance methods estimate dose based on minute changes to tooth enamel caused from radiation exposures^2^. Humans^3^, rodents^4^, dogs^5^, reindeer^6^ and cows^6^ are among the many species in which ESR analyses of tooth enamel have been used to estimate radiation dose. Harshman *et al*.^1^ determined the detection limit of their ESR analyses was 1.8 Gray (Gy). All boar in the cataract study, except one, had a dose based on the ESR method less than the detection limit^1^. These results provided confidence that the upper-bound lifetime dose of the boar, based on the ESR method, was < 1.8 Gy.

The large number of samples below the ESR detection limit prevented a thorough statistical analysis of cataract prevalence and severity as a function of chronic radiation exposure. Therefore, lifetime dose to each boar was also estimated based on external dose from the animal’s contaminated environment, combined with internal dose from ^134+137^Cesium (Cs) activity concentrations within each animal’s muscle tissues. The internal dose arises from ingestion of Cs contaminated food and water.

We conservatively selected values in the lifetime dose calculation to be at a ***plausible upper-bound***. Selecting values to conservatively maximize an animal’s dose is often used as a first step in a multi-tiered risk analysis^8,9,10^. If effects are not observed at the conservative, upper-bound dose then researchers can be confident that the same effect was not observed in animals exposed to the lesser, realistic, but unknown dose. Lifetime dose was estimated for each animal’s whole-body, not organ-specific to the ocular lens.

*External Dose from Ambient Dose Rates*

To estimate a plausible upper-bound, lifetime external dose for each boar we used dose rate distribution maps of the Fukushima Prefecture generated by the Japanese government^11^. The maps are produced using highly sensitive radiation detectors installed on aircraft flown annually in a grid pattern over the contaminated areas. MEXT dose rate maps are useful because they (1) show spatial and temporal changes in dose rates (µSv h^-1^ at 1 m height above ground surface), (2) include dose from ^134^Cs, ^137^Cs and naturally occurring radionuclides, and (3) are geographically interactive such that the precise location of each boar’s capture site can be plotted on the MEXT dose rate maps.

To maximize external dose estimates for each animal we selected a MEXT dose rate map corresponding in time to when the animal was estimated to be < 6 months of age. For example, the MEXT data from 2016 were used for boar that were between 26 and 30 weeks of age at time of capture, and MEXT data from 2013 were used for boar that were > 220 weeks of age at capture (**Table S-1**). This assured the dose rate from external exposure was pertinent when the animal was young and the dose from ^134^Cs was properly incorporated into the dose rate estimation. Exposure to ^134^Cs decreased over the life of the older boars due to its physical decay (^134^Cs half-life = 2.1 years).

Each boar’s trap site was plotted on the appropriate year-specific MEXT map and the highest dose rate within a 5-km distance of the trap site was used. We thus purposely over-estimated each boar’s external dose rate by: (1) selecting the highest dose rate value from an area larger than the typical boar’s home range (5-20 km^2^), (2) selecting a MEXT map that was generated when the animal was young and ^134^Cs levels the greatest, and (3) multiplying the conservatively chosen dose rate (µSv h^-1^) by the animal’s age, further magnifying the conservatism to derive a “plausible upper-bound lifetime external dose”.

No attempt was made to convert H*(10) ambient dose rates (µSv) as presented on the MEXT maps to absorbed dose (µGy) via energy specific air kerma rates^12^. Those corrections are relatively small (e.g. 0.92)^13^. Therefore, the ambient dose rate readings from MEXT (µSv h^-1^) were changed directly to absorbed dose rates (µGy h^-1^) appropriate for non-humans.

One limitation of using the MEXT data for these calculations is the data are truncated at “> 19 µSv h^-1^” for the highest dose rate category mapped. Even though the most contaminated sites (i.e. > 19 µSv h^-1^) occur at a relatively low frequency of < 3%^14^, using a MEXT value of 19 µSv h^-1^ would tend to lower the lifetime upper-bound dose estimates. Therefore, we examined data from several dose rate ground surveys^14,15,16,17^ and opted to replace “>19 µSv h^-1^” with 40 µSv h^-1^ for the upper level dose rate category in the calculations.

*Internal Dose from Tissue Analyses for Cesium Radioisotopes*

Dose rates from internal contamination were based on activity concentrations (Bq kg^-1^, fresh wt.) of ^134^Cs and ^137^Cs in muscle tissue taken from each boar carcass. Activity concentrations were measured at the Institute of Environmental Radioactivity (Fukushima University) by gamma spectrometry using HPGe semiconductor detectors with calibrated standards of the same geometry as the samples. Energy-specific dose conversion factors were obtained from the ERICA Assessment Tool, version 1.2^10^ and used to convert activity concentrations (Bq kg^-1^) in boar muscle tissues to internal dose rates (µGy h^-1^). To estimate lifetime dose, internal dose rates (µGy h^-1^) at the time of capture were multiplied by the age of the animal. However, multiplying activity concentrations of ^134^Cs in muscle tissues by the animal’s age underestimates dose rates to older animals due to ^134^Cs decay during the animal’s lifetime. To obtain a more reasonable lifetime dose estimate from internal contamination, particularly for older boar, we back-calculated the increased contribution of ^134^Cs during the earlier period of each animal’s life. The back-calculated ^134^Cs corrections resulted in an additional internal dose that contributed as little as 9% to the total lifetime dose from ^134^Cs for a young animal (26 weeks of age at capture), and as much as 120% increase in internal dose from ^134^Cs to the oldest boar (> 220 weeks). The back-calculation increased the conservatism of the dose estimations and helped achieve a plausible upper-bound estimate of lifetime dose.

**RESULTS**

The combined external and internal dose estimates produced “plausible upper-bound, lifetime dose” for each Japanese boar (Supplementary Table S1).

**Supplementary Table S1.** Plausible upper-bound, lifetime dose for Fukushima boar following initial dose determination via electron spin resonance (ESR). This method combined external and internal dose for both ^134^Cs and ^137^Cs to determine values lower than 1.8 Gy that were undetected by ESR.

| Boar ID | Trap site GPS (East) | Trap site GPS (North) | Boar age (weeks) | Date of MEXT data used | MEXT µSv/h @ 1m height, maximum in 5 km of trap site | MEXT lifetime, 134+137Cs Ext. dose; mGy | ^134^Cs (Bq/kg, from tissue analyses) | ^134^Cs, build-up corrected, internal lifetime dose, mGy | ^137^Cs (Bq/kg, from tissue analyses) | ^137^Cs Internal lifetime dose, mGy | Plausible upper-bound lifetime dose (Int + Ext, 134+137) mGy |
| --- | --- | --- | --- | --- | --- | --- | --- | --- | --- | --- | --- |
| Ba20170605 | 140.9889 | 37.4804 | 47-52 | Nov 2015 | 40* | 330 | 118 | 0.6 | 1,050 | 2.70 | 333 |
| Ba20170608 | 140.9967 | 37.4892 | 56 | Nov 2015 | 9.5 | 89 | 19 | 0.1 | 159 | 0.5 | 90 |
| Ba20170609 | 140.9262 | 37.4653 | > 220 | Nov 2013 | 40 | 1,512 | 617 | 28.6 | 4,710 | 55.6 | 1596 |
| Bb20170609 | 141.0061 | 37.4761 | 47-52 | Nov 2015 | 40 | 336 | 65 | 0.4 | 550 | 1.4 | 338 |
| Ba20170615 | 140.4999 | 37.7612 | > 220 | Nov 2013 | 1 | 38 | 25 | 1.2 | 194 | 2.3 | 42 |
| Ba20170616 | 141.0061 | 37.4761 | 47-52 | Nov 2015 | 40 | 336 | 94 | 0.5 | 769 | 2.0 | 338 |
| Bb20170616 | 141.0084 | 37.4922 | 62 | Nov 2015 | 3.8 | 40 | 43 | 0.3 | 318 | 1.0 | 41 |
| Ba20170617 | 140.9849 | 37.7564 | 26 | Nov 2016 | 0.2 | 1 | 4 | 0.01 | 30 | 0.04 | 1 |
| Ba20170620 | 140.9232 | 37.4653 | 62 | Nov 2015 | 40 | 417 | 508 | 3.6 | 3,920 | 12.7 | 433 |
| Ba20170623 | 140.9404 | 37.5011 | 127 | Nov 2014 | 40 | 853 | 105 | 1.9 | 826 | 5.5 | 861 |
| Bb20170623 | 140.7830 | 37.5971 | 88-106 | Nov 2015 | 3.8 | 62 | 269 | 3.4 | 2,070 | 10.5 | 76 |
| Ba20170627 | 140.9235 | 37.4644 | 62 | Nov 2015 | 40 | 417 | 502 | 3.5 | 4,070 | 13.2 | 433 |
| Ba20170704 | 140.5569 | 37.2804 | 56-62 | Nov 2015 | 40 | 403 | 525 | 3.6 | 4,020 | 12.6 | 419 |
| Ba20170717 | 140.9594 | 37.5057 | 57-61 | Nov 2015 | 40 | 403 | 129 | 0.9 | 1,020 | 3.2 | 407 |
| Bb20170717 | 140.9695 | 37.4878 | 127 | Nov 2014 | 40 | 853 | 124 | 2.2 | 1,040 | 6.9 | 863 |
| Bc20170717 | 141.0025 | 37.4768 | 127 | Nov 2014 | 40 | 853 | 84 | 1.5 | 711 | 4.7 | 860 |
| Ba20170724 | 140.9835 | 37.4809 | 62 | Nov 2015 | 40 | 471 | 192 | 1.4 | 1220 | 4.0 | 422 |

*All values of 40 were substituted for the truncated upper limit of “> 19” used within the MEXT data set

**REFERENCES**

1. Harshman, A. & Johnson, T. Dose reconstruction using electron paramagnetic resonance dosimetry on tooth enamel from wild boar living in the Fukushima Exclusion Zone. *Health Physics* **116(6),** 799-806 (2019).
2. Serezhenkov, V., Moroz, I., Klevezal, G., & Vanin, A. Estimation of accumulated dose of radiation by the method of ESR-spectrometry of dental enamel of mammals. *Applied Radiation and Isotopes* **47(11-12),** 1321–1328 (1996).
3. Fattibene, P. & Callens, F. EPR dosimetry with tooth enamel: a review. *Applied Radiation and Isotopes* **68(11),** 2033–2116 (2010).
4. Khan, R.F., Rink, W. & Boreham, D. Biophysical dose measurement using electron paramagnetic resonance in rodent teeth. *Applied Radiation and Isotopes* **59(2-3),** 189–196 (2003).
5. Khan, R.F., Pekar, J., Rink, W. & Boreham, D. Retrospective radiation dosimetry using electron paramagnetic resonance in canine dental enamel. *Applied Radiation and Isotopes* **62(2),** 173–179 (2005).
6. Klevezal, G., Serezhenkov, V. & Bakhur, A. Relationships between ESR-evaluated doses estimated from enamel and activity of radionuclides in bone and teeth of reindeer. *Applied Radiation and Isotopes* **50(3),** 567–572 (1999).
7. Toyoda, S., *et al*. Gamma-ray dose response of ESR signals in tooth enamel of cows and mice in comparison with human teeth. *Radiation Measurements* **37(4-5)** 341–346 (2003).
8. Suter, G.W., II, R.A. Efroymson, B.E. Sample, & Jones, D.S. *Ecological Risk Assessment for Contaminated Sites*. Lewis Publishers, Boca Raton, Florida. (2000).
9. Copplestone, D., S, *et al*. Impact Assessment of Ionising Radiation on Wildlife. Bristol, UK, Environment Agency, **32(37)** 222pp. (2001)
10. Brown, J.E., *et al*. A new version of the ERICA tool to facilitate impact assessments of radioactivity on wild plants and animals. *Journal of Environmental Radioactivity* **153,** 141-148 (2016).
11. MEXT, 2014. *Extension Site of Distribution Map of Radiation Dose*, etc. http://ramap.jmc. or.jp/map/eng/, Accessed date: 03 March 2019.
12. ICRP. Conversion coefficients for use in radiological protection against external radiation. In: *International Council on Radiological Protection Publication* 74 Ann. ICRP 26, 1-205 (1996).
13. Kubota, Y. *et al.* Estimation of absorbed radiation dose rates in wild rodents inhabiting a site severely contaminated by the Fukushima Dai-ichi nuclear power plant accident. *Journal of Environmental Radioactivity* **142,** 124-131 (2015).
14. Mikami, S., *et al*. The air dose rate around the Fukushima Dai-ichi Nuclear Power Plant: its spatial characteristics and temporal changes until December 2012. *Journal of Environmental Radioactivity* **139,** 250-259 (2015).
15. Andoh, M., *et al*. Measurements of air dose rates over a wide area around the Fukushima Dai-ichi Nuclear Power Plant through a series of car-borne surveys. *Journal of Environmental Radioactivity* **139,** 266-280 (2015).
16. Sanada, Y., Orita, T., Torii, T. Temporal variation of dose rate distribution around the Fukushima Daiichi nuclear power station using unmanned helicopter. *Applied Radiation and Isotopes* **118,** 308-316 (2016).
17. Andoh, M., Yamamoto, H., Kanno, T., Saito, K.. Measurement of ambient dose equivalent rates by walk survey around Fukushima Dai-ichi Nuclear Power Plant using KURAMA-II until 2016. *Journal of Environmental Radioactivity* **190-191,** 111-121. (2018).
